# A Bayesian Assignment Method for Ambiguous Bisulfite Short Reads

**DOI:** 10.1371/journal.pone.0151826

**Published:** 2016-03-24

**Authors:** Hong Tran, Xiaowei Wu, Saima Tithi, Ming-an Sun, Hehuang Xie, Liqing Zhang

**Affiliations:** 1 Department of Computer Science, Virginia Polytechnic Institute and State University (Virginia Tech), Blacksburg, Virginia, United States of America; 2 Department of Statistics, Virginia Polytechnic Institute and State University (Virginia Tech), Blacksburg, Virginia, United States of America; 3 Virginia Bioinformatics Institute, Virginia Polytechnic Institute and State University (Virginia Tech), Blacksburg, Virginia, United States of America; 4 Department of Biological Sciences, Virginia Polytechnic Institute and State University(Virginia Tech), Blacksburg, Virginia, United States of America; Inc, UNITED STATES

## Abstract

DNA methylation is an epigenetic modification critical for normal development and diseases. The determination of genome-wide DNA methylation at single-nucleotide resolution is made possible by sequencing bisulfite treated DNA with next generation high-throughput sequencing. However, aligning bisulfite short reads to a reference genome remains challenging as only a limited proportion of them (around 50–70%) can be aligned uniquely; a significant proportion, known as multireads, are mapped to multiple locations and thus discarded from downstream analyses, causing financial waste and biased methylation inference. To address this issue, we develop a Bayesian model that assigns multireads to their most likely locations based on the posterior probability derived from information hidden in uniquely aligned reads. Analyses of both simulated data and real hairpin bisulfite sequencing data show that our method can effectively assign approximately 70% of the multireads to their best locations with up to 90% accuracy, leading to a significant increase in the overall mapping efficiency. Moreover, the assignment model shows robust performance with low coverage depth, making it particularly attractive considering the prohibitive cost of bisulfite sequencing. Additionally, results show that longer reads help improve the performance of the assignment model. The assignment model is also robust to varying degrees of methylation and varying sequencing error rates. Finally, incorporating prior knowledge on mutation rate and context specific methylation level into the assignment model increases inference accuracy. The assignment model is implemented in the BAM-ABS package and freely available at https://github.com/zhanglabvt/BAM_ABS.

## Introduction

DNA methylation is the addition of a methyl group (CH3) at the 5th carbon position of the cytosine ring. Cytosine methylation frequently occurs in the sequence context of 5’CG3’ (also called a CpG dinucleotide) in mammalian DNA. Non-CpG methylation at CpH dinucleotides (where H = C, T or A) has been reported in some specific cell types, such as adult brain tissues [1] and stem cells [2]. DNA methylation leads to condensed chromatin and transcriptionally silences genes on the inactive X chromosome, imprinted loci, and parasitic DNAs [3]. It is also a major contributor to the generation of disease-causing germ-line mutations and somatic mutations that cause cancer [4]. The determination of DNA methylation is crucial for the understanding of phenotype differences among cells or tissues during development and disease.

With the advance of next generation sequencing technology, characterization of genome-wide DNA methylation at single-nucleotide resolution is made possible by whole-genome bisulfite sequencing. After bisulfite treatment of DNA, unmethylated Cs are converted to Uracils by sodium bisulfite, then in the downstream PCR/sequencing step, Uracils are read as Ts, whereas methylated Cs remain unchanged. Subsequent mapping of the short reads to a reference genome allows inference of methylated vs. unmethylated Cs. Several factors make bisulfite short reads (BS-reads) more complicated to map than regular short reads. First, due to how BS-reads are generated, after PCR amplification, up to four strands might be produced from one genomic region. The search space is therefore significantly increased. Second, sequence complexity is reduced, as most of the unmethylated Cs are changed into Ts. Third, C to T mapping is asymmetric. The T in the bisulfite reads could be mapped to either C or T in the reference genome but not vice versa [5]. Despite the introduction of several bisulfite short read alignment tools (e.g., Bismark [6], BSMAP [5], BS-Seeker [7], and Batmeth [8]), the mapping efficiency of BS-reads remains very low, that is, a high percentage of BS-reads, nearly 50% are either mapped to multiple genomic locations (called “multireads” or “ambiguous” reads) or unmapped [9].

Most BS-read mapping programs, for instance, Bismark [6], BS-Seeker [7], and Batmeth [8], convert both the genome and the reads to a three-letter alphabet accounting for the C-to-T or G-to-A mismatches caused by bisulfite conversion before applying a regular short read mapper such as Bowtie [10] or BWA [11]. However, due to reduced complexity in C-to-T and G-to-A conversion, this simple strategy causes a greatly increased proportion of reads to be aligned to multiple genomic locations with similar scores, i.e., multireads. The routine practice is to exclude all the multireads and unmapped reads from downstream analyses. This practice leads to not only bias in estimating methylation levels but also financial waste.

**Fig 1:**
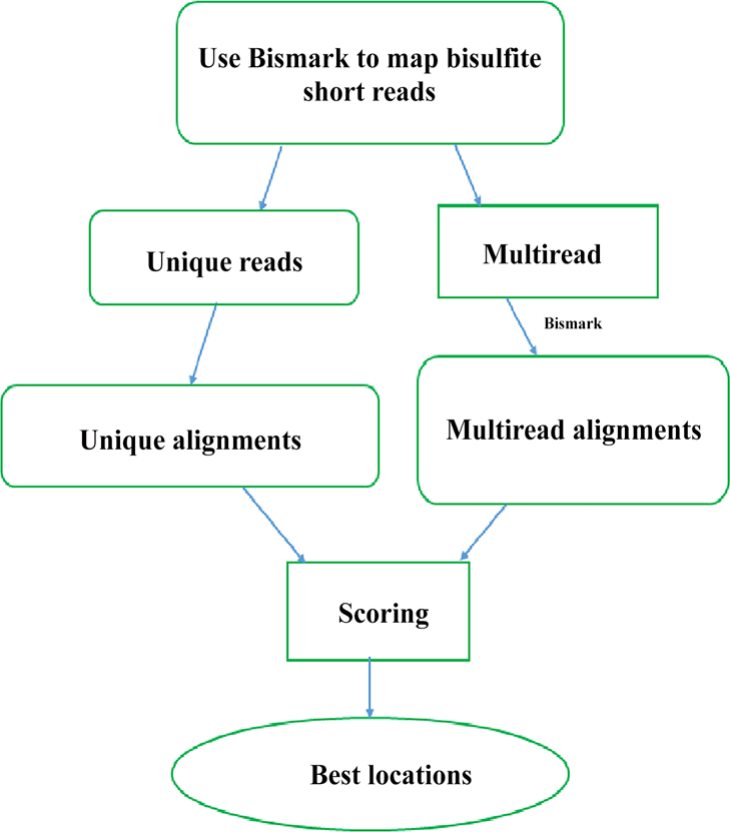
Pipeline for assigning multireads to the best locations.

In this paper, we present a Bayesian statistical method to solve the multiread mapping problem so that a great number of ambiguously mapped reads can be allocated to the most probable genomic locations, thus improving the overall mapping efficiency. To this end, we use the mismatch and methylation profiles between multireads and genomic locations, taking advantage of the information gleaned from unique read alignments, prior knowledge of single nucleotide polymorphisms (SNPs), and context-specific methylation levels at the regions, to assign each multiread to the best location according to the highest posterior probability. Our assignment framework involves two stages. First, we use Bismark—a popular BS-reads mapper [12] to map the BS-reads, and from the mapping results, compile all the multireads with their competing locations as well as all the unique reads overlapping with the multireads. The second stage is refinement, during which we deploy the proposed Bayesian model to assign each multiread to the most likely genomic location (Fig 1). We use both simulated data and real data generated with hairpin bisulfite sequencing strategy to evaluate performance of our Bayesian assignment method.

## Materials and Methods

### Posterior probability calculation

Suppose, for a given multiread *X* with length *K*, that there are *T* competing genomic locations, indexed by *t* = 1,⋯,*T*, and that the multiread is mapped with similar fidelity (e.g., equal or similar number of mismatches). For genomic location *t*, we use *M*_*k*_ to denote the observed base of the multiread *X* at position *k* (*k* = 1,⋯,*K*) of the genomic location and *R*_*k*_ to denote the reference base (i.e., the base that the reference genome has) at that position. The overlapping unique reads are defined as reads that are uniquely mapped with high mapping qualities (usually with MAQ scores greater than 30) and also overlapped with a multiread’s mapped location. Assuming that there are *r* such unique reads, we use *D*_*k*_ = {*d*_1*k*_, *d*_2*k*_, …*d*_*rk*_} to denote the observed bases of overlapping unique reads at position *k*. Given the multiread and genomic location *t*, the observed data consist of two mismatch profiles, one between the reference genome and the multiread, the other between the reference genome and all the overlapping unique reads. We want to compute the posterior probability of observing *M*_*k*_ given *D*_*k*_, *P*(*M*_*k*_|*D*_*k*_), based on which decision is made on assigning the multiread.

Applying Bayes’ Theorem,

$$P\left(M_{k}|D_{k}\right)=\frac{\pi \left(M_{k}\right)P\left(D_{k}|M_{k}\right)}{\pi \left(M_{k}\right)P\left(D_{k}|M_{k}\right)+\pi \left({\bar{M}}_{k}\right)P\left(D_{k}|{\bar{M}}_{k}\right)},$$

where *π*(*M*_*k*_) is the prior probability of observing base *M*_*k*_ and *P*(*D*_*k*_|*M*_*k*_) is the likelihood of observing the overlapping unique reads at position k given the observed *M*_*k*_. In practice, we would also like to incorporate the reference information *R*_*k*_ into the prior to help improve the inference accuracy. Replacing *π*(*M*_*k*_), $\pi \left({\bar{M}}_{k}\right)$ with *π*(*M*_*k*_|*R*_*k*_), $\pi \left({\bar{M}}_{k}|R_{k}\right)$, respectively, and assuming that conditioning on *M*_*k*_, *D*_*k*_ is independent of *R*_*k*_, we may write the posterior probability as

$$P\left(M_{k}|D_{k},R_{k}\right)=\frac{\pi \left(M_{k}|R_{k}\right)P\left(D_{k}|M_{k}\right)}{\pi \left(M_{k}|R_{k}\right)P\left(D_{k}|M_{k}\right)+\pi \left({\bar{M}}_{k}|R_{k}\right)P\left(D_{k}|{\bar{M}}_{k}\right)}.$$

How the prior probability *π*(*M*_*k*_|*R*_*k*_) is computed is given in the next section.

Since the likelihood *P*(*D*_*k*_|*M*_*k*_), as the product of all *P*(*d*_*jk*_|*M*_*k*_) for *j* = 1…*r*, is directly related to the number of overlapping unique reads: the more reads, the smaller likelihood, we calculate *P*(*D*_*k*_|*M*_*k*_) in an average sense instead of using the usual joint probability definition to avoid this bias. Thus we write the likelihood in terms of the base quality of the multiread and unique reads as

$$P\left(D_{k}|M_{k}\right)=\frac{\sum_{j=1}^{r}\;P\left(d_{jk}|M_{k}\right)}{r}$$

where

$$P\left(d_{jk}|M_{k}\right)=\left\{ \begin{array}{ll} 1-\varepsilon_{jk}-\varepsilon_{k}+\varepsilon_{jk}\times \varepsilon_{k}, & \text{if}\;d_{jk}=M_{k}\\ \varepsilon_{jk}+\varepsilon_{k}-\varepsilon_{jk}\times \varepsilon_{k}, & \text{if}\;d_{jk}\neq M_{k} \end{array}\right.,$$

and *ε*_*jk*_ is the probability of observing a base miscall in the *j*th unique read at position *k*, *ε*_*k*_ is the probability of observing a base miscall in the multiread at position *k*. It is easy to see that the above calculation follows the general addition rule of probability, that is *P*(*A*∪*B*) = *P*(*A*) + *P*(*B*) − *P*(*A*∩*B*). Here, *A* represents the event of having a sequencing error in the *j*th unique read at position *k*, and *B* represents the event of having a sequencing error at the multiread base *M*_*k*_. Given sequencing errors occur independently in unique reads and in multireads, i.e., *P*(*A*∩*B*) = *P*(*A*)*P*(*B*), replacing *P*(*A*) with *ε*_*jk*_, and *P*(*B*) with *ε*_*k*_ then results in the expression of *P*(*d*_*jk*_|*M*_*k*_).

Finally we calculate the posterior probability of observing the multiread *X* at genomic location *t* by

$$P\left(X|D,R\right)=\prod_{k=1}^{K}P\left(M_{k}|D_{k},{\text{R}}_{\text{k}}\right),$$

where ***D*** = {*D*_1_, *D*_2_,…,*D*_*K*_} denotes the set of all observed bases from the overlapping unique reads at positions 1,2,⋯,*K*. The genomic location with the highest posterior probability is chosen, and an assignment score *S* for the read is calculated by taking the log odds of the posterior probabilities at the best location and at the next best location:

$$S=\text{log}\frac{P(X|D)\;\text{at best location}}{P(X|D)\;\text{at next best location}}.$$ (1)

To assign a multiread, we need to determine a cutoff score *S*_0_. Users can choose a cutoff score suitable to their needs. If a multiread has an assignment score *S* ≥ *S*_0_, the read is considered as “assignable” and will be assigned to the best location, otherwise, the read will be labeled as “unassignable”. We conducted experiments to determine a cutoff score *S*_0_. Experiments show that the Bayesian assignment model achieves good performance when *S* is set between 0.005 to 6. We set *S*_0_ to be 0.05 in simulated data and 0.2 in real data. In real data, the sequence coverage is not uniform across the entire genome and some genomic loci may not be covered by any uniquely mapped read. We will assign a multiread to a location that has more unique reads. To increase inference accuracy, we raise the cut-off in real data to 0.2 and achieved a reasonable efficiency in the multiread assignment.

### Prior probability calculation

Given the reference genome, the mutation rate of the organism, the observed multiread sequence, and knowledge on context-specific methylation levels, we can infer the underlying process and compute *π*(*M*_*k*_|*R*_*k*_), the prior probability of observing multiread base *M*_*k*_ given the reference genome base *R*_*k*_ at position *k*. For example, according to NCBI dbSNP [13], transitions are twice as frequent as transversions in many species, such as humans and mice. Also, studies have shown that the methylation rate is about 0.80 at CpG whereas 0.05 at CH (H∈{A,T,C}) in mammals [14]. Such information can be incorporated to compute *π*(*M*_*k*_|*R*_*k*_). To illustrate, suppose that the reference genome has a base C at one position of the genomic location that the multiread is aligned to, then there are four possible cases:

1) observing A in the multiread

In this case, we conclude that there is only a C to A mutation occurring and the prior probability of observing A in the multiread given C in the reference genome is

$$\pi (M_{k}|R_{k})=P(\text{C to A mutation}).$$

2) observing C in the multiread

In this case, we conclude that no mutation occurs and the C is methylated. The prior probability of observing C in the multiread given C in the reference genome is

$$\pi (M_{k}|R_{k})=[1-P(\text{mutation})]\times P(\text{methylation}).$$

3) observing G in the multiread

In this case, we conclude that there is only a C to G mutation occurring and the prior probability of observing G in the multiread given C in the reference genome is

$$\pi (M_{k}|R_{k})=P(\text{C to G mutation}).$$

4) observing T in the multiread

In this case, we conclude that either there is a C to T mutation occurring or there is no mutation and the C in the reference genome is unmethylated and converted to T after bisulfite treatment. Therefore the prior probability of observing T in the multiread given C in the reference genome is the sum of the probabilities of the two disjoint events and can be expressed as

$$\pi (M_{k}|R_{k})=P(\text{C to T mutation})+[1-P(\text{mutation})]\times \left[1-P(\text{methylation})\right].$$

The probability of C methylation *P*(methylation) depends on the sequence context, that is, if the next base in the multiread is G, the probability of C methylation is higher than that if the next base is H (H∈{A,T,C}). The probability of mutation can be computed similarly as in previous methods [15], [16]. For example, if we assume that the SNP rate in the human genome is 0.001 and that the reference allele is C at position *k*, the prior probabilities of C to A mutation and C to G mutation are 0.00025 and 0.00025, respectively, whereas the prior probability of C to T mutation is 0.0005 and the prior probability of C to C (i.e., no mutation) is 0.999. All other cases are illustrated in tables in S1 Table and S2 Table. In a later section of simulation study and real data analysis, we will also consider the “without” prior option, that is, using a uniform prior (equal probabilities for observing different bases on *M*_*k*_) and make a comparison to illustrate the advantage of using a prior in the assignment model.

### Bisulfite short read simulation

We aim to generate BS-reads that closely mimic the bisulfite conversion experiment. The simulated data consist of BS-reads generated from the human genome (hg19) and the mouse genome (mm10). First, we randomly assigned a mutation rate of 0.001 to every base in the reference genome, i.e., we randomly changed 0.1% of all current bases in the reference genomes to other bases. As transitions are twice as frequent as transversions, we assigned a higher probability for C↔T and G↔A mutations than other mutations, e.g., P(C ↔ T) = 0.0005 while P(C ↔ A) = P(C ↔ G) = 0.00025. Second, we randomly assigned a methylation rate to every cytosine in both strands of each chromosome after introducing mutations. We varied the methylation probability at CpG (i.e., 70%, 75%, 80%, 85%, 90%) while maintaining methylation probability at CH (H∈{A,T,C}) 0.5%. To illustrate, we randomly converted C to T at 99.5% of all CH sites and converted C to T at 30%, 25%, 20%, 15% or 10% of all CpG sites to generate different data sets. After introducing both mutation and methylation, we randomly generated short reads with different read lengths for each data (51 bp, 76 bp, and 101 bp) for each data from the converted reference genome. Finally, we extracted quality score strings from three real datasets SRR980327 (read length = 51 bp), SRR342553 (read length = 76 bp), and SRR921765 (read length = 101 bp) generated by the Illumnia-HiSeq 2000 platform (data downloaded from NCBI’s short read archive [17]) and simulated sequence errors according to the per-base error probabilities of all reads from these datasets. All reads were generated in a directional manner, i.e., only from the top strands of the genome. We simulated 3,000, 40,000, and 100,000 short reads for each methylation probability parameter with varying read lengths.

We used Bismark [6] to align simulated BS-reads and collected all ambiguous reads or multireads. Most of the multireads have two or three mapped genomic locations in both simulated and real data (S1 Fig). In this paper, we only examined directional data. However, undirectional data will be addressed similarly, since only methylation and SNP information of uniquely mapped reads from the same DNA strand as a multiread is incorporated in the scoring model.

An important and practical question is how much coverage is required for accurate assignment of multireads using our model. To address this problem, for each location that multireads are aligned to, we generated different numbers (i.e., 3x, 5x, 10x, 25x, and 30x) of overlapping unique reads to mimic different depths of coverage. We then introduced sequencing errors for the generated reads using base quality scores from the real data. These reads are treated as overlapping unique reads in our Bayesian assignment model. A detailed pipeline for generating BS-reads and overlapping unique reads is illustrated in S2 Fig.

### Real data from hairpin bisulfite sequencing

**Fig 2:**
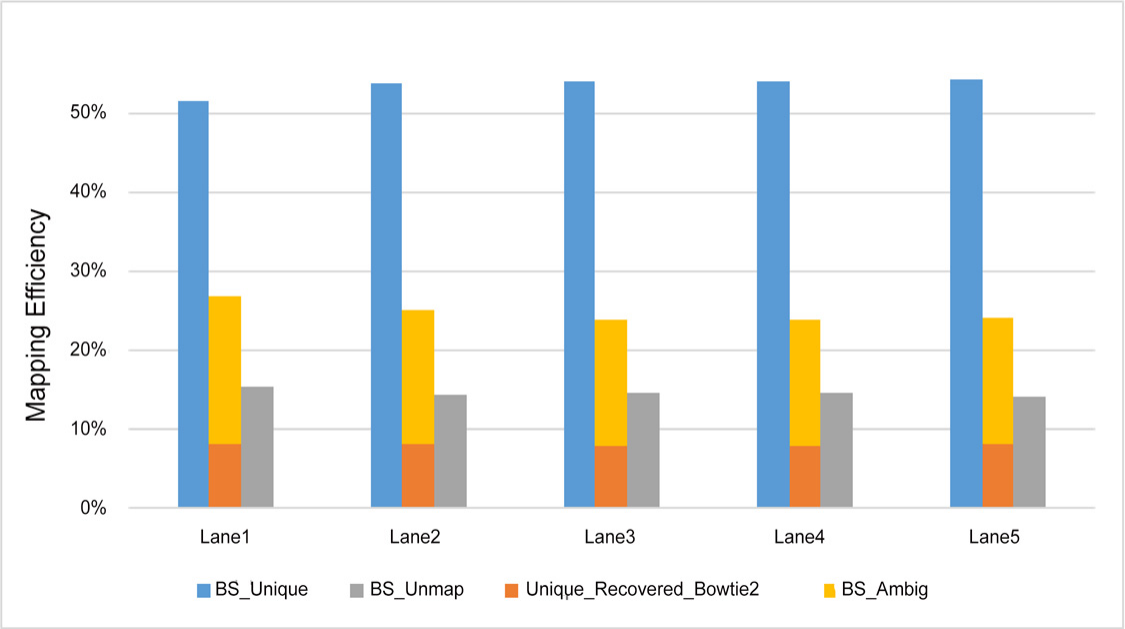
Mapping efficiency using Bismark on the mouse embryonic stem cell data for different categories, uniquely mapped reads (blue), multireads (yellow), and unmapped reads (grey). The orange bar is the percentage of multireads that become uniquely mapped with Bowtie2 after recovery to their original sequences using the hairpin bisulfite sequencing technique.

To validate our model on real data, we used the genome-scale hairpin bisulfite sequencing data for mouse embryonic stem cell (ESC) (NCBI’s SRA accession number: GSM1173118) produced in our previous study [18]. The hairpin data are from one sample but generated in five different sequencing lanes (labeled as Lane1, Lane2, Lane3, Lane4, Lane5). In brief, genomic DNA was extracted and then sonicated into fragments of around 200 bp. Then, the DNA fragments were ligated to the biotinylated hairpin and Illumina sequencing adaptors simultaneously. Following the streptavidin-capture and bisulfite PCR, the fragments linked to both the hairpin adaptor and Illumina sequencing adaptor were amplified for high-throughput paired-end sequencing using Illumina HiSeq 2000 platform. After purification, size selection of 400–600-bp fragments was conducted with LabChip XT DNA Assay (Caliper) to yield longer sequences that are more amenable for unambiguous mapping to the reference sequence. The reads are of 101 bp in length. Unlike traditional bisulfite sequencing methods, which are non-invertible, the hairpin technology allows for recovery of the original sequences; therefore, hairpin data can be used to evaluate the mapping efficiency of BS-reads. The hairpin sequencing approach generates methylation data for two DNA strands simultaneously by putting a linking adaptor between Watson and Crick strands and then using PCR and paired-end technology to sequence short reads [19] (S3 Fig). The resulting sequences give paired strands so that the original untreated sequences can be recovered. Taking advantage of this ability, we used Bismark [20] with default parameters and Bowtie2 [10] option (command:./bismark—path_to_bowtie <path to Bowtie2 folder>—bowtie2—ambiguous <path to Reference genome folder> <input_short_reads.fastq>) to map approximately 308 million reads generated with genome-scale hairpin bisulfite sequencing. Bismark [6] mapped ~ 50% reads uniquely and 25% ambiguously (Fig 2). We collected all the ambiguous reads, recovered their original sequences, and used Bowtie2 [10] with default parameters (command:./bowtie2 -x <reference.fa> -U <input_short_reads.fastq> -S <output.sam>) to map the original sequences. Here the mapping results of recovered sequences are used as the gold standard to validate our Bayesian assignment model. To ensure the quality of the gold standard, we used only those reads with mapping quality score ≥30. As a measure of the goodness of alignment, mapping quality score is a non-negative integer Q = -10 log10p, where p is an estimate of the probability that the alignment does not correspond to the read's true point of origin. Mapping quality is sometimes abbreviated MAPQ. Approximately 48% of the recovered reads were mapped uniquely and also satisfied our mapping quality requirement, and thus were used to validate our model (Fig 2). We randomly sampled 1% and 10% of the reads, respectively, from Lane1, Lane2, Lane3, Lane4 and Lane5. We created ten replicates from 1% random sampling and ten other replicates from 10% random sampling for each of the five lanes. Therefore, we had 100 samples altogether, to generate some of the statistics.

### Real data from regular bisulfite sequencing

Although the hairpin bisulfite sequencing data seem ideal as the gold standard from real data, there is still concern that it might differ in some way from data produced by the regular bisulfite sequencing procedure. Therefore, we also applied our assignment model to another real data produced by the regular whole-genome bisulfite sequencing for the human brain (NCBI’s SRA accession number: GSM1163695). The human brain data include ten datasets. The DNA bisulfite short read sequences are directional. Each dataset contains around 100 million single-end bisulfite reads for the human frontal cortex. The reads have conventional base call qualities that are Illumina HiSeq 2000 encoded Phred values (Phred64) and have been trimmed to 101 bps. We used Bismark with default parameters to map all the short reads from the ten datasets. Bismark mapped ~75% reads uniquely and ~8% ambiguously. We then used these uniquely mapped reads as “gold standard” to assess the performance of the model. The idea is to shorten these reads so that the original uniquely mapped reads become ambiguously mapped reads, then we apply our model to assign these reads and use the original mapped location as the correct answer to evaluate the assignment accuracy of our model. Specifically, we randomly sampled 1% of the uniquely mapped reads from the ten datasets and trimmed the reads to shorter ones (i.e., 10 bp shorter than original short reads). After applying Bismark to the trimmed reads, ~50% were uniquely mapped and ~5% multireads. We used our Bayesian model to assign the location of these trimmed multireads and compared the assigned locations with their originally mapped locations.

## Results

### Mapping efficiency improvement for simulated data and real data

**Fig 3:**
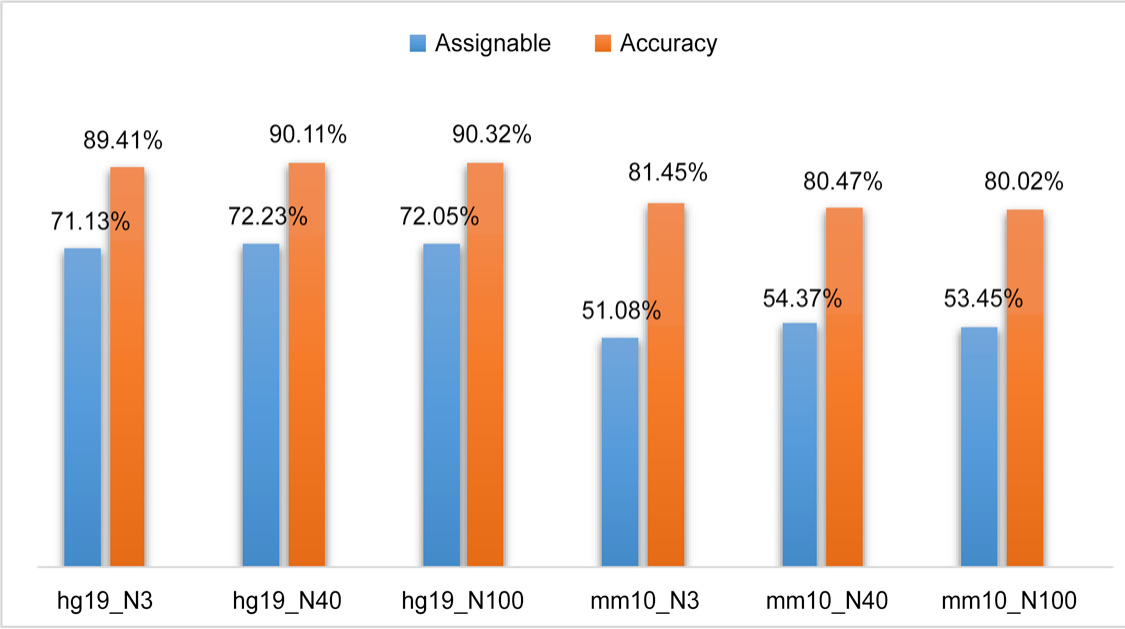
Percentages of assignable multireads and accuracy rates of the assigned multireads on six simulated bisulfite datasets generated from the human reference and the mouse reference with read length = 76 bp and CG = 20% (20% of all CG -cytosines are converted into thymines) and CH = 99.5% (99.5% of all CH -cytosines are converted into thymines) and mutation rate of 0.1% at 30x coverage. hg19_N3, hg19_N40, and hg19_N100 denote the datasets with 3k, 40k, and 100k simulated reads respectively for humans; mm10_N3, mm10_N40, and mm10_N100 denote the datasets with 3k, 40k, and 100k simulated reads respectively for mice. All remaining figures use the same notations.

We simulated 3,000, 40,000, and 100,000 BS-reads for both the human genome and the mouse genome with the setting of read length = 76 bp, CG = 20% (20% of all CG -cytosines are converted into thymines), CH = 99.5% (H can be A, T, or G, 99.5% of all CH -cytosines are converted into thymines), and mutation rate of 0.1% at 30x coverage. We then applied the Bayesian assignment model to score the ambiguously mapped BS-reads and assigned them to their best locations based on the log likelihood ratio *S* (Eq 1). For human BS-reads, the model was able to assign ~ 72% of the multireads to their best locations with an assignment accuracy rate of ~90% for all three datasets (Fig 3). The accuracy rate was defined as the percentage of correctly assigned multireads, i.e., the ratio of the number of accurately assigned multireads to the number assigned multireads. For mouse BS-reads, the model was able to assign approximately 53% of all the multireads with an accuracy rate of 80%. Both percentages of assignable multireads and accuracy rates for the mouse data were lower than those for the human. This is likely due to the fact that there are more CTs or TCs in the mouse genome than in the human genome (26.37% vs. 23.87%), consequently, with bisulfite treatment, the mouse genomic DNAs are expected to have a higher frequency of TT posing more challenges to multiread assignment.

A major challenge in testing the performance of multiread assignment methods on real data is a lack of ground truth for where multireads should be assigned to in the real data. To examine the performance of our Bayesian assignment model on real data, we took advantage of the genome-scale hairpin bisulfite sequencing technique developed recently [21] that allows us to recover the bisulfite converted reads to their original sequences. We assume that once multireads are recovered to their original sequences and these original sequences are mapped to unique locations, the unique locations are indeed true locations. To ensure this assumption to be largely held, we consider only those multireads that are mapped with high mapping quality.

**Fig 4:**
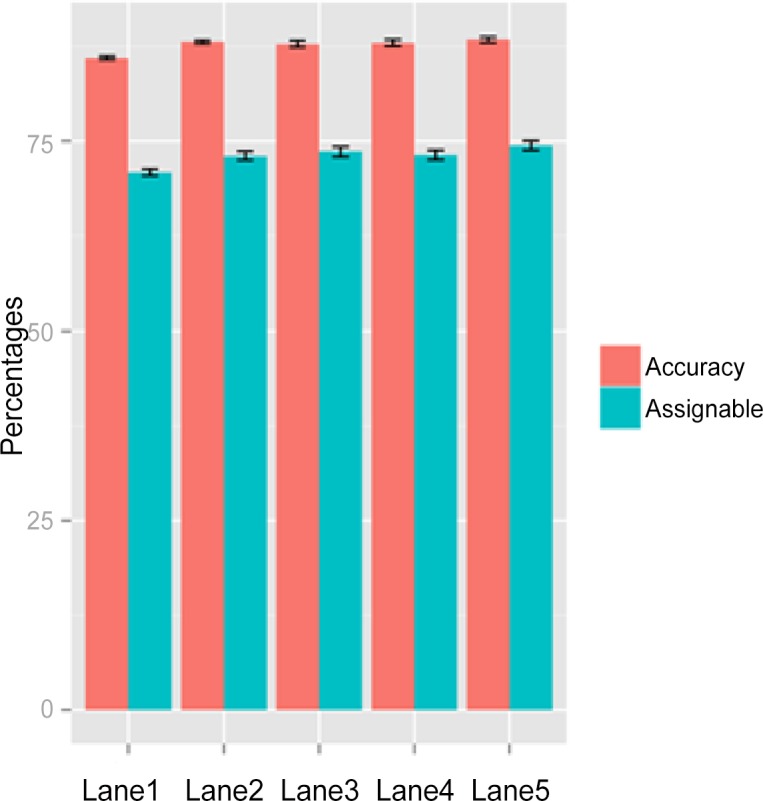
Accuracy rates of assigned multireads and percentages of assignable multireads on ten replicates from 1% random samples from five genome-wide hairpin bisulfite sequencing datasets from mouse ESC. The black bar shows the standard deviation.

The genome-scale hairpin bisulfite sequencing data for mouse ESC were generated in five sequencing lanes with the Illumina sequencing platforms. For data generated from each of the five lanes, we randomly sampled 1% of the reads and created ten samples per dataset. With assignment score cut-off of 0.2, in the range of reasonable cut-off point by experiment, 74% of the multireads were assigned to their best locations with ~88% accuracy rates (Fig 4). Standard deviations across ten replicates were small, from 0.23–0.42% and from 0.46–0.66% in accuracy rates and assignable percentages, respectively. Thus, 1% random samples were representative of the five datasets.

**Fig 5:**
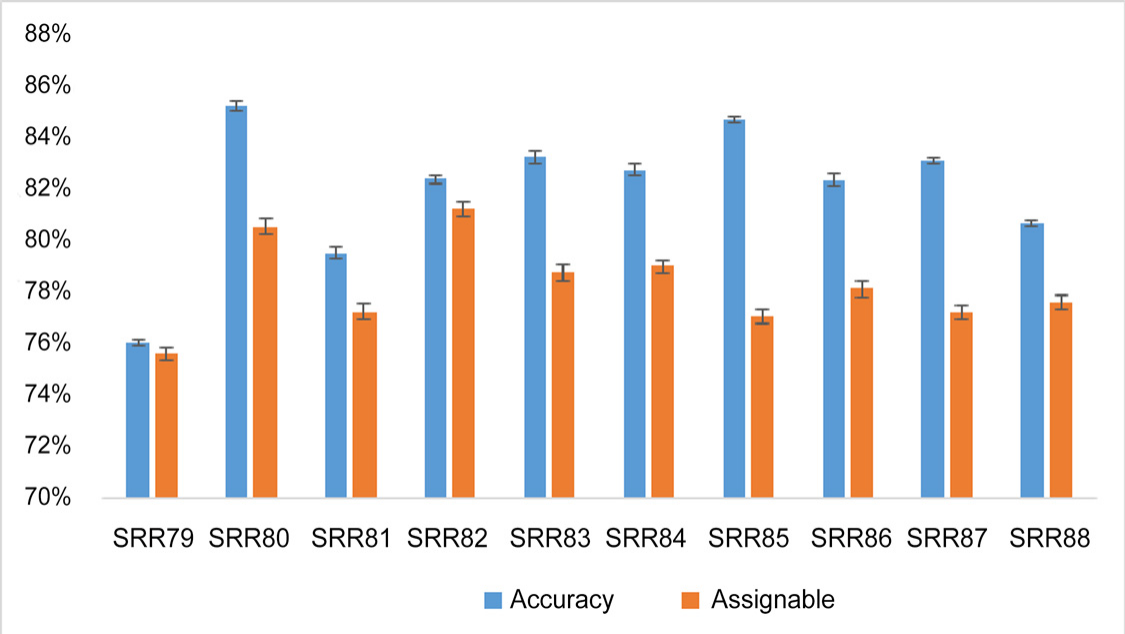
Accuracy rates of assigned multireads and percentages of assignable multireads on ten replicates from 1% random samples from ten genome-wide bisulfite sequencing datasets from human frontal cortex (SRA accession number GSM1163695). The black bar shows the standard deviation.

For human brain whole-genome bisulfite sequencing data, we randomly sampled 1% of the uniquely mapped reads from ten datasets, shortened them so that they “degraded” from previously uniquely mapped reads to multireads. Our model assigned ~75–81% of the multireads to their best locations with ~76–85% accuracy rates (Fig 5), therefore, showing similar performance results to that for hairpin sequencing data.

### Effect of coverage depth and with/without prior

Table 1 shows the effect of sequence coverage on the performance of the model, with and without priors for simulated data. For the simulated human data, the percentage of assignable multireads tends to increase with the coverage depth, and expectedly, the assignment error rate decreases. Compared to simple assignment without a prior, that is, only using observed unique reads to assign multireads, considering prior probability *π*(*M*_*k*_|*R*_*k*_) leads to better performance in the model, with much lower error rates (9%-11% compared to 22%-33% for without a prior), although the percentage of assignable multireads decreases at the same time. When the comparison is converted to error rates per read, it is clear that incorporating priors in the method increases the mapping accuracy, with the error rate per read decreasing from 0.01% to 0.005% for the 3x coverage data, and 0.007% to 0.003% for the 30x coverage. The simulated mouse data show a similar pattern, except, in general, has lower percentages of assignable multireads and higher error rates.

**Table 1:** The percentage of assignable multireads and the error rate (ratio of the # of reads assigned to wrong locations to the # of reads that were assigned) as a function of coverage depth and with or without priors for simulated data.

Coverage depth	Without prior		With prior	
	Assignable rate (%)	Error rate (%)	Assignable rate (%)	Error rate (%)
hg19_N40				
3x	96.23	32.55	67.20	10.5
5x	98.10	32.48	69.34	9.96
10x	99.43	27.32	70.55	9.23
25x	99.58	21.95	71.63	9.01
30x	99.37	21.54	72.23	9.00
mm10_N40				
3x	92.56	44.55	49.18	20.68
5x	96.74	44.34	52.44	20.89
10x	98.98	40.67	54.96	19.98
25x	99.43	36.68	54.96	19.81
30x	99.41	36.48	54.37	19.53

For hairpin bisulfite sequencing data, when including prior probabilities, even though the percentages of assignable multireads reduce, the error rates per read decrease (Table 2). For example, error rates reduce from 0.00043% to 0.00035% and from 0.00025% to 0.00020% in Lane5_1 and Lane2_10 respectively. Therefore, incorporating priors in the method increases inference accuracy. These results are consistent with simulation results. Compared with simulation results, the accuracy rate improvement in real data is smaller.

**Table 2:** Assignable rates and error rates for assigning multireads with and without priors on 1% and 10% random samples from five genome-wide hairpin bisulfite sequencing datasets from mouse ESC (without priors refers to only using observed unique reads to assign multireads).

Sample ID	Without prior			With prior		
	Assignable rate(%)	Error rate (%)	Error per read (%)	Assignable rate (%)	Error rate (%)	Error per read (%)
Lane1_1	72.17	17.50	0.00043	70.97	14.01	0.00035
Lane1_10	72.27	18.30	0.00004	71.27	13.90	0.00003
Lane2_1	74.60	14.74	0.00239	73.61	11.35	0.00187
Lane2_10	74.67	15.53	0.00025	73.27	12.13	0.00020
Lane3_1	74.44	15.12	0.00275	73.54	12.78	0.00235
Lane3_10	74.54	14.58	0.00026	73.61	12.17	0.00022
Lane4_1	73.24	15.07	0.00282	72.27	12.39	0.00235
Lane4_10	74.35	14.79	0.00027	73.32	12.21	0.00023
Lane5_1	74.76	14.27	0.00251	73.77	12.12	0.00216
Lane5_10	74.23	14.44	0.00025	73.38	12.02	0.00021

We also determined the effect of read coverage on the performance of the assignment model using hairpin sequencing data. Specifically, coverage depth refers to the number of unique reads that overlap with multireads and thus can be used for inference. Table 3 shows that as coverage depth increases from 6x to 40x, assignment accuracy increases slightly from 85.92% to 86% in Lane1 and the percentage of assignable reads decreases slightly from 70.9% to 70.82% in Lane1, both at a lower rate than in the simulation study.

**Table 3:** Coverage effect on model performance for 1% random samples from the five hairpin datasets.

**Coverage**	**Lane 1**		**Lane 2**		**Lane3**	
	Assignable rate (%)	Accuracy rate (%)	Assignable rate (%)	Accuracy rate (%)	Assignable rate (%)	Accuracy rate (%)
**6x**	70.90	85.92	73.62	88.65	73.41	87.14
**10x**	70.92	85.92	73.63	88.68	73.37	87.17
**20x**	70.90	85.95	73.58	88.69	73.45	87.22
**130x**	70.90	85.99	73.47	88.71	73.42	87.25
**40x**	70.82	86.00	73.47	88.71	73.53	87.33
**Coverage**	**Lane 4**		**Lane 5**			
	Assignable rate (%)	Accuracy rate (%)	Assignable rate (%)	Accuracy rate (%)		
**6x**	72.23	87.322	73.73	87.79		
**10x**	72.27	87.329	73.77	87.83		
**20x**	72.28	87.464	73.70	87.84		
**30x**	72.16	87.481	73.73	87.84		
**40x**	72.19	87.505	73.74	87.86		

Noteworthy is that the model performs well even with low coverage, for both simulated data and real data. Taken together, the robust performance of the assignment model towards low coverage data makes the model particularly applicable to the current whole genome bisulfite sequencing data (many at 10x coverage).

### Effect of read length

**Fig 6:**
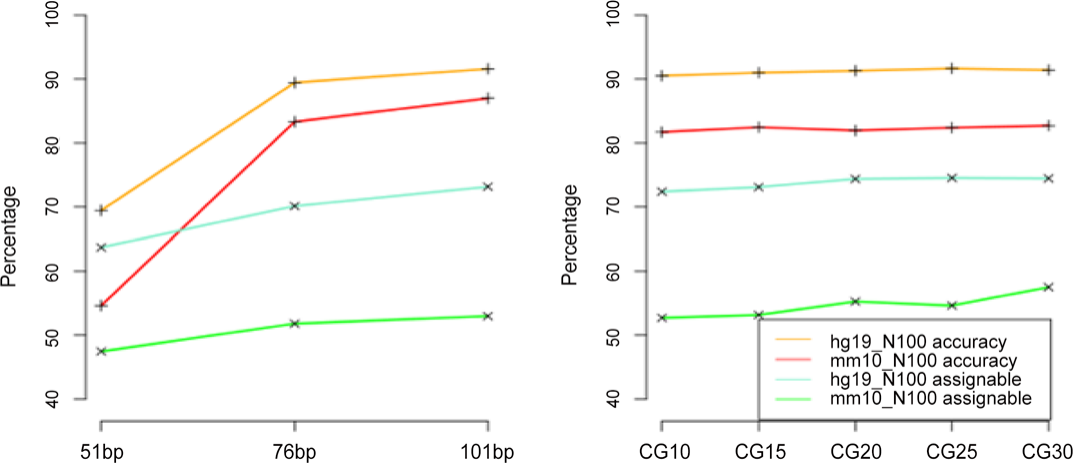
Effect of read length (left panel) and methylation rates at CpG s (right panel, CG10 refers to a methylation rate of 90% at CpGs) on the percentage of assignable multireads and assignment accuracy rates for simulated data generated from hg19 and mm10 at 30x coverage.

To examine the effect of read length on the performance of the Bayesian assignment model, we simulated BS-reads with three read lengths, 51bp, 76bp, and 101bp. All simulated data (3K, 40K, and 100K reads for humans and mice) show similar patterns and only data with 100K BS-reads were used to demonstrate for brevity. Fig 6 (left panel) shows that for both human and mouse data, as read length increases, the accuracy rate of assigned multireads to their true locations increases as well as the percentage of assignable multireads. The percentage of increase in accuracy rate is much higher for read lengths increasing from 51bp to 76bp than from 76bp to 101bp.

**Fig 7:**
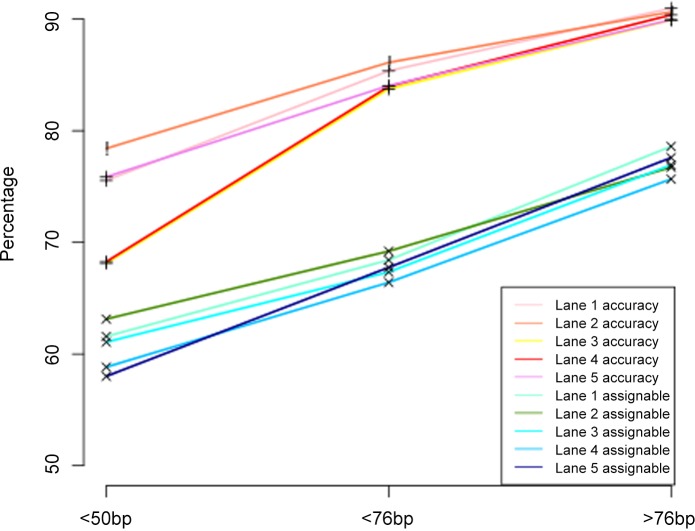
Effect of read length on accuracy rates and percentages of assignable multireads on 1% random samples from five genome-wide hairpin bisulfite sequencing datasets from ESC.

In our real data analysis, the hairpin bisulfite sequencing data contain reads with different lengths (S4 Fig). This enabled us to determine the effect of read length on our model performance. Reads were classified into 3 groups: short, with read length ≤ 50 bp, moderate, with read length between 50–76 bp, and long, with read length > 76 bp. Fig 7 shows that as read length increases, assignable percentages of multireads increase as well as accuracy rates on 1% random samples from the five whole-genome mouse hairpin ESC data. Reads in the long group have highest accuracy rates, around 90% and highest assignable rates, around 75%. Notably, more than a 10% increase in accuracy was observed from the short and moderate groups (i.e., accuracy rate in Lane1 dataset jumps from 75.55% to 85.36%, approximately 10% increase in accuracy).

### Effect of methylation rate at CpGs

As methylation may vary as a function of genomic regions, developmental stages, tissues, species, and so on [14] [22], it is important to examine how the multiread assignment model is affected by varying methylation rates. We therefore simulated data with different methylation rates (70%, 75%, 80%, 85%, 90%) at CpGs and applied the Bayesian model to assign the multireads in the data. Fig 6 (right panel) shows that both the percentage of assignable multireads and assignment accuracy rate change only slightly with respect to different methylation rates, indicating that the method is robust to changes in methylation rates.

### Effect of sequencing errors

To examine the effect of sequencing error on the assignment model, we simulated data with different sequencing error rates ranging from 0.002% to 3%. Table 4 shows that as sequencing error increases, for both humans and mice, accuracy rate of multiread assignment decreases. However the percentage of assignable ambiguous reads remains similar. Comparatively, sequencing error has a bigger impact on the mouse data than on the human data.

**Table 4:** Effect of sequencing errors on the percentage of assignable reads for simulated data generated from hg19 and mm10 at 30x coverage.

Sequencing error	Accuracy rate (%)		Assignable rate (%)	
	hg19_N40	mm10_N40	hg19_N40	mm10_N40
0.002%	99.31	99.36	71.10	55.05
0.005%	99.12	98.68	71.15	55.19
0.015%	98.97	98.10	71.50	56.60
0.045%	98.62	97.37	71.31	50.87
0.150%	96.97	93.60	71.54	52.23
0.500%	96.31	89.82	72.21	56.06
1.500%	95.30	85.40	72.04	52.28
3.000%	93.23	82.16	72.81	55.56

## Discussion

The whole genome bisulfite sequencing technique allows for determination of C methylation at the whole genome scale and with single nucleotide resolution. Though considered to be the gold standard for characterizing DNA methylation, its high cost has limited its application to large research laboratories. To make the situation worse, the mapping efficiency of existing tools has been low, mostly 50–70% as compared to over 95% in regular short reads mapping [12]. A large proportion of reads, known as multireads, are routinely discarded from downstream analysis, leading to both biased methylation inference and financial loss. To address the problem, we propose a Bayesian assignment model to help determine the most likely locations the multireads should be mapped to. Results show that the model is effective and can be used to increase the number of uniquely mapped read, and thus allows users to make the best use of the data possible.

Our analysis demonstrates that read length shows a much bigger positive impact on the model performance for real data than for simulated data: both the percentage of assignable reads and the assignment accuracy rate increase much more with read length increase in real data (Fig 7) than in simulated data (Fig 6). This is likely because reads from real data carry more information than simulated reads giving the assignment model more power to differentiate among the competing locations of multireads, and thus lead to better performance in real data. We note that real whole genome bisulfite sequencing experiments usually generate reads with 100bp or longer. Even after ends trimming, these reads are mostly longer than 76bp. The results here suggest that, with real data, the assignment model is capable of recovering 14–20% of the multireads to their true locations (Fig 2), and these reads can be included in downstream analysis to provide more comprehensive information on methylation at the genome level. It might be interesting to conduct a comprehensive survey to examine how these reads that are routinely thrown away affect the downstream inference were they included in the downstream analysis.

Due to the high cost of whole genome bisulfite sequencing, the depth of sequencing coverage is often low, approximately 10X for many experiments. This poses an additional challenge to downstream analyses such as methylation calling and variant calling. For example, Bis-SNP, a program that does methylation calling and SNP calling for bisulfite sequencing data, requires an average of 30X coverage for correctly calling 96% of the SNPs [23]. Our results demonstrate that even with low coverage of ~5X-10X, the Bayesian scoring model performs well and is stable (Tables 1 and 3).

Our Bayesian scoring model enables a high proportion of multireads to be mapped to unique locations, which in turn increases the overall amount of sequence data suitable for the downstream methylation inference. An interesting issue to examine is whether methylation ratios are affected as a result of changes in the compositions of reads. Thus, we took a set of 50,000 multireads and ~500,000 uniquely mapped reads overlapping with these multireads and another set of ~550,000 uniquely mapped reads in these regions from the human whole-genome bisulfite sequencing data (SRA accession number SRX306253, GSM1163695, see methods for details) and used Bismark for methylation calling. The methylation ratios at CpG sites were very similar between the two datasets. We also took a set of 100,000 multireads and ~300,000 uniquely mapped reads and another set of ~400,000 uniquely mapped reads around these regions and did the same analysis. The methylation ratios were still similar but as expected there were more CpG sites covered in the former dataset. Taken together, the results suggest it depends on data coverage and percentages of multireads. Specifically, CpG methylation ratios are expected to stay similar if the coverage is low, however, more CpG methylation sites will be covered. On the other hand, if the coverage is high, CpG methylation ratios are expected to be more accurate and more CpG sites will be covered. Again, the advantage of multiread mapping is to gain valuable information from “unusable” data by traditional mappers, which benefits the subsequent calling procedure and downstream analysis.

Results for both simulated data and real data (Tables 1 and 2) show that incorporating prior knowledge such as mutation rates and context specific methylation levels into the assignment model helps improve the accuracy of the assignment. Moreover, for organisms without such prior information, the assignment model can still provide robust assignment, especially reflected by the real data. Comparatively, it is clear that information gleaned from uniquely mapped reads plays a more important role in correctly assigning multireads.

A common problem in the development of tools for bisulfite short read mapping is the lack of a gold standard. We addressed this by taking advantage of the hairpin bisulfite sequencing data that allows the recovery of the original reads (see S3 Fig for the mechanism of read recovery), and assuming that the unique locations that recovered reads are mapped to are true locations. Although we required a high mapping quality (≥30), it is still possible that some of the true locations are false positives. However, the consistency shown between simulated data and real data suggests that even if there are false positives in the gold standard, the number should be very low. Another concern for using hairpin bisulfite sequencing data is that its characteristics might be different from those of the regular bisulfite sequencing data. However, our model performance on regular bisulfite sequencing data is very similar to that on hairpin sequencing data, suggesting that the hairpin sequencing data is representative and can serve as gold standard for real data.

## Conclusion

A major problem in mapping bisulfite short reads is the high percentage of multireads caused by bisulfite conversion. To our knowledge, no program is devoted to address this problem. Here we present a Bayesian model to assign multireads to the best possible locations. Simulation and real data results show that our assignment method is effective in mapping multireads with high accuracy. We investigated several factors that might affect the model performance, including methylation level, coverage, sequencing error, and read length. More specifically, methylation level has little effect, whereas sequencing errors have a negative impact on model performance. Increasing depth of coverage and read length will increase the accuracy of assigning multireads. The model performs quite well even with low read coverage. Therefore, our scoring method can be used to effectively improve the mapping results of bisulfite sequencing data.

## Supporting Information

S1 Fig Histogram of number of genomic locations Bismark found for multireads in simulated data (left) and in real hairpin data (right). (TIF)

S2 Fig Pipeline for generating bisulfite short reads, multireads, and overlap unique reads. (TIF)

S3 Fig Example of bisulfite-treated hairpin PCR sequencing with recovery of the original sequence. (TIF)

S4 Fig Histograms of read length from Lane1, Lane2, Lane3, Lane4, and Lane5 hairpin data. (TIF)

S1 Table Prior calculation. (PDF)

S2 Table Prior probabilities of all possible cases of alignments on the reverse direction. (PDF)
