# Supplementary figures and images for: A Bayesian Assignment Method for Ambiguous Bisulfite Short Reads
Source: PLoS One. 2016 Mar 24;11(3):e0151826. doi: 10.1371/journal.pone.0151826 (PMC4806927; doi:10.1371/journal.pone.0151826)

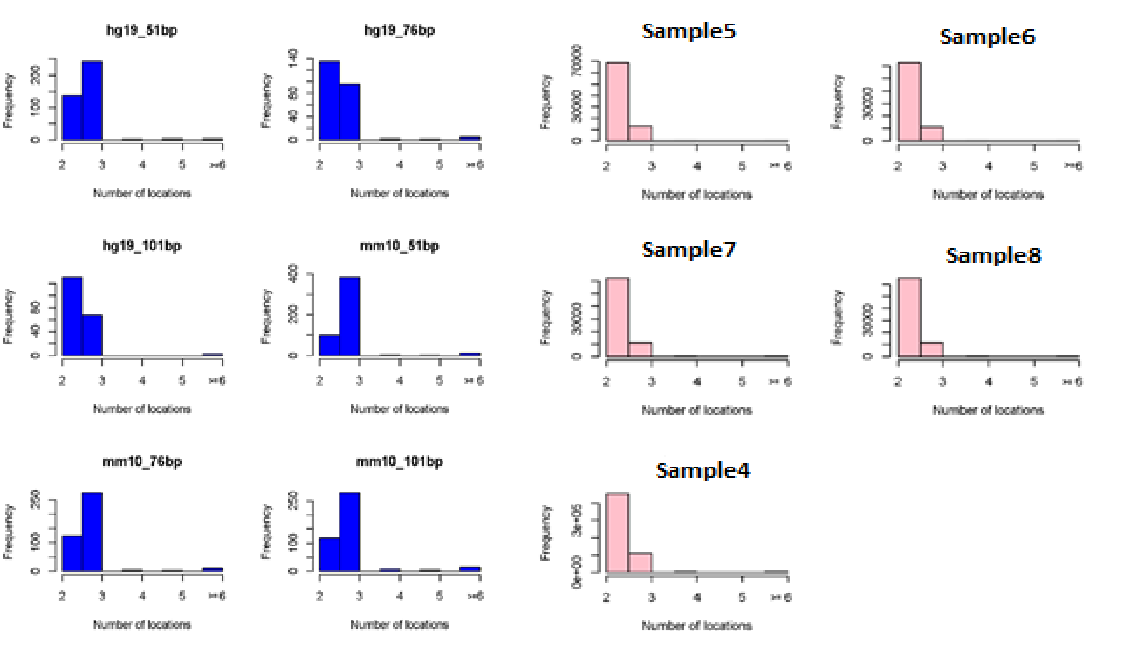

Supplement: S1 Fig — (TIF) [file pone.0151826.s001.tif]

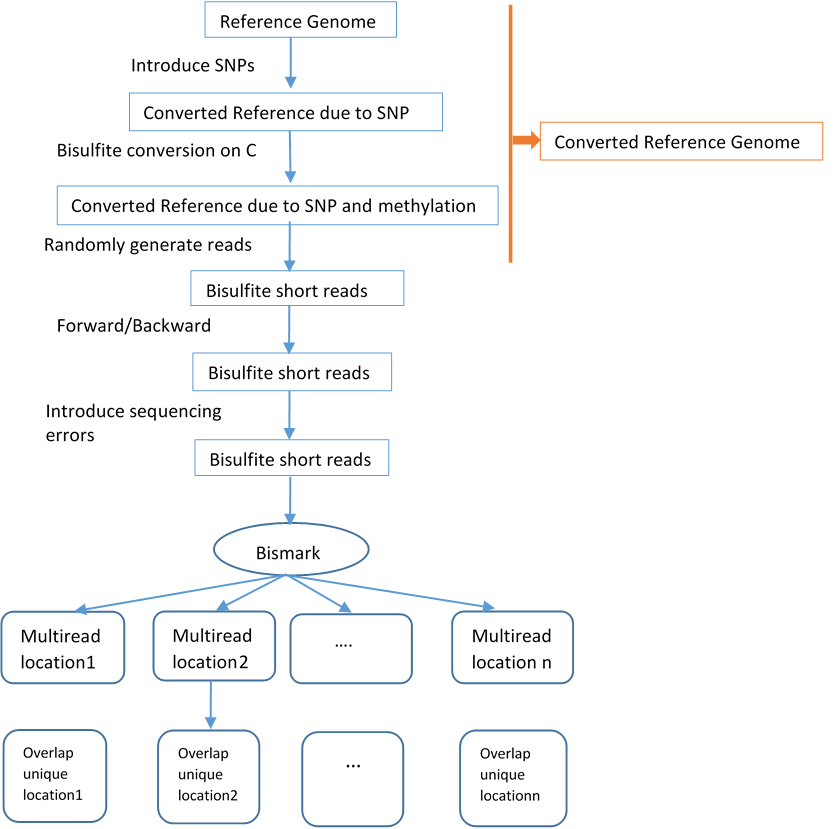

Supplement: S2 Fig — (TIF) [file pone.0151826.s002.tif]

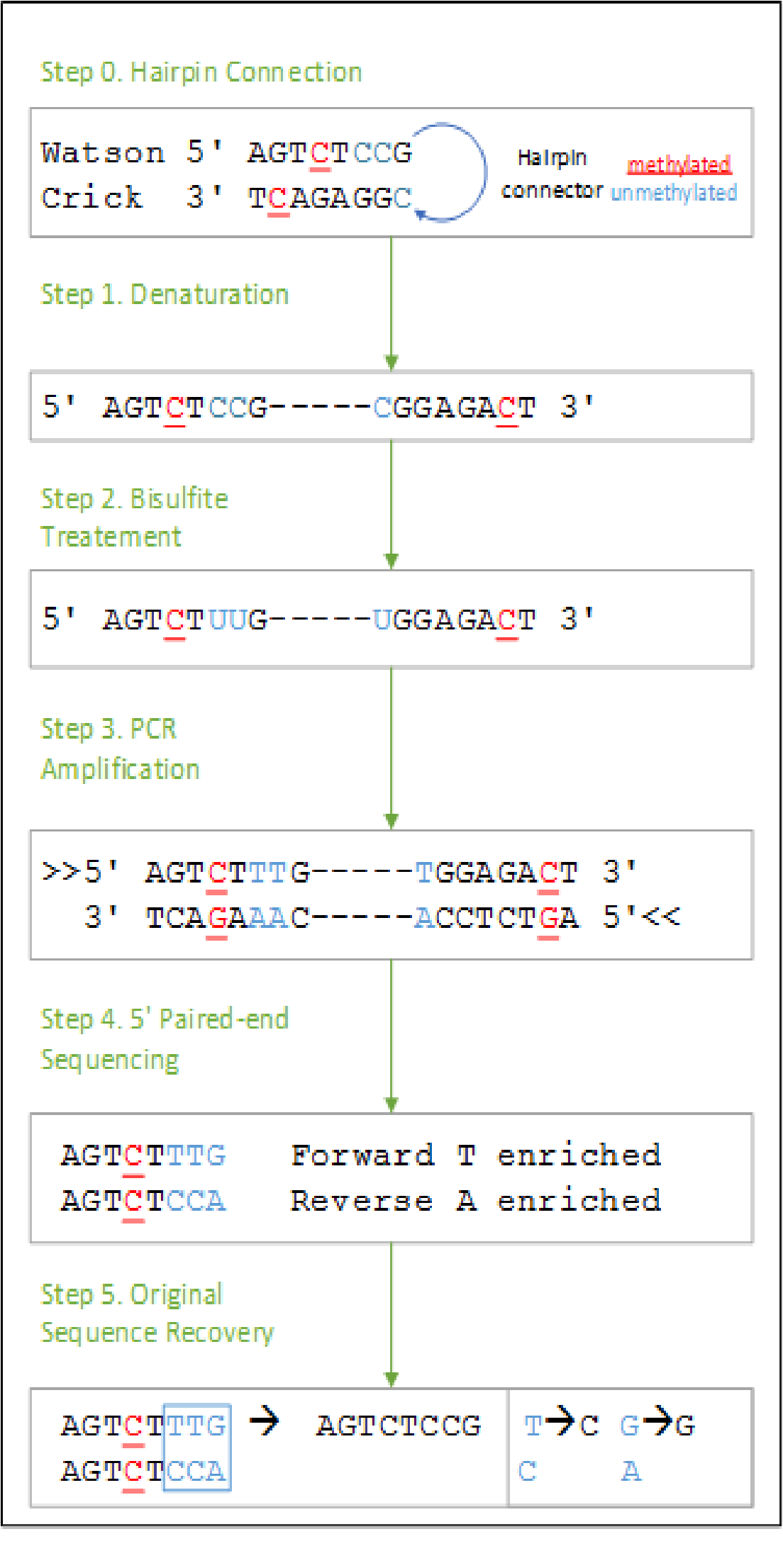

Supplement: S3 Fig — (TIF) [file pone.0151826.s003.tif]

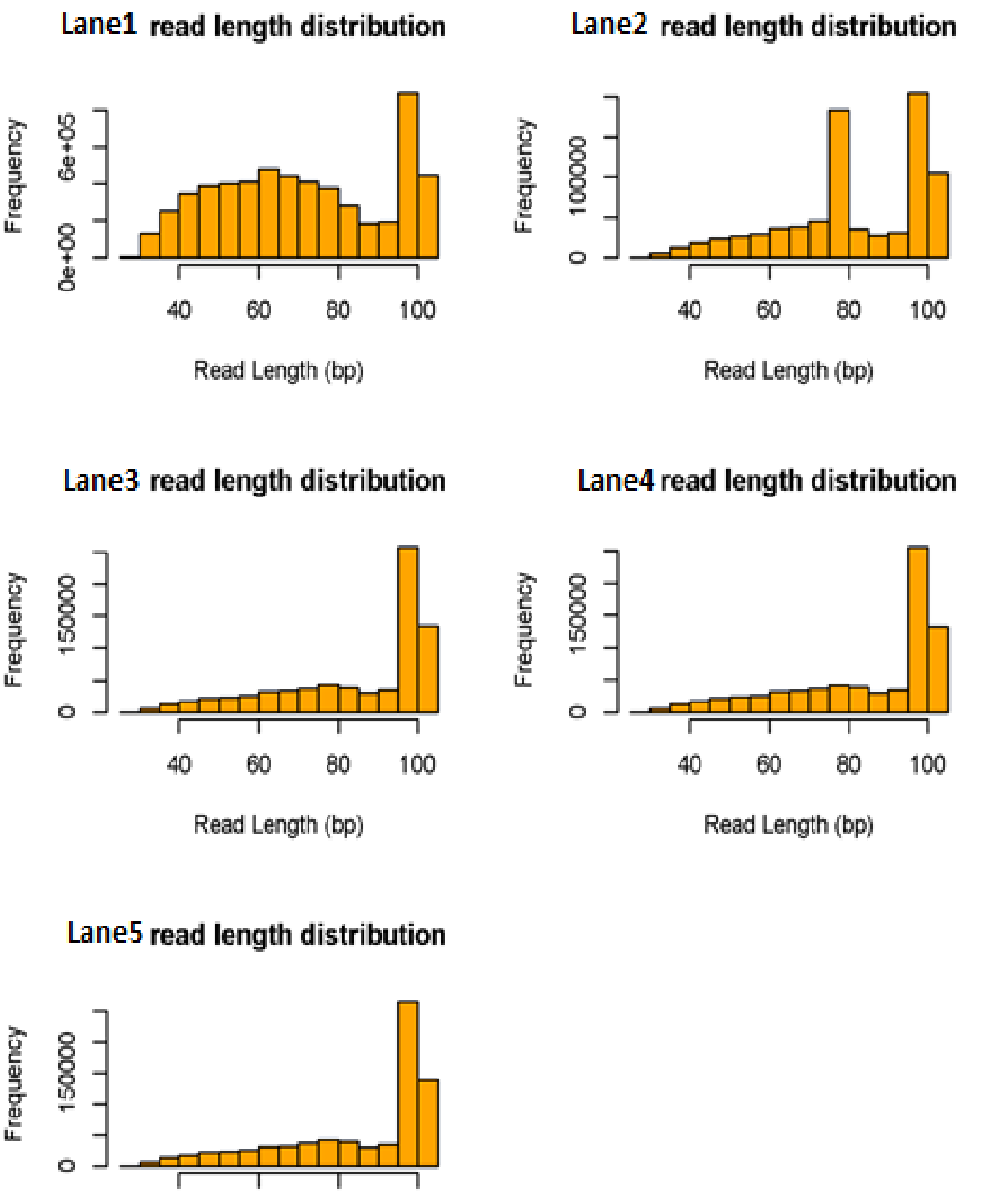

Supplement: S4 Fig — (TIF) [file pone.0151826.s004.tif]
