# Supplementary material for: A Bayesian Assignment Method for Ambiguous Bisulfite Short Reads
Source: PLoS One. 2016 Mar 24;11(3):e0151826. doi: 10.1371/journal.pone.0151826 (PMC4806927; doi:10.1371/journal.pone.0151826)
Supplement: S1 Table — (PDF) [file pone.0151826.s005.pdf]

Prior probabilities of all possible cases of alignments on the forward direction

Notation:

Pr(me) is the probability of methylation event occurring at a position
Pr(SNP) is the probability of mutation event occurring at a position

Pr(AB) is the probability of A to B mutation event occurring at a position, i.e. Pr(AT) is the probability of A on the reference genome changes to T on the multiread.

Bases in green are observed bases, in black are unobserved. Cs/Gs in red indicate methylated Cs/Gs, in blue unmethylated Cs/Gs.

Table 1a: Prior probabilities at A reference genome of forward alignments

Reference base	**A**	**A**	**A**	**A**
Unobserved	**A**	**C**	**G**	**T**/**C**
Multiread base	**A**	**C**	**G**	**T**
Inference	No mutation	A to C mutation and methylated C	A to G mutation	A to T mutation or A to C mutation and unmethylated C
Prior	1-Pr(SNP)	Pr(AC)xPr(me)	Pr(AG)	Pr(AT)+Pr(AC)x[1-Pr(me)]

Note: 1-Pr(SNP) + Pr(AC)xPr(me)+ Pr(AG)+ Pr(AT)+Pr(AC)x[1-Pr(me)]=1 (sum of all priors is 1)

Table 1b: Prior probabilities at C reference genome of forward alignments

Reference base	**C**	**C**	**C**	**C**
Unobserved	**A**	**C**	**G**	**T**/**C**
Multiread base	**A**	**C**	**G**	**T**
Inference	C to A mutation	No mutation and methylated C	C to G mutation	C to T mutation or no mutation and unmethylated C
Prior	Pr(CA)	[1-Pr(SNP)]*Pr(me)	Pr(CG)	Pr(CT)+[1-Pr(SNP)]x[1-Pr(me)]

Table 1c: Prior probabilities at G reference genome of forward alignments

Reference base	**G**	**G**	**G**	**G**
Unobserved	**A**	**C**	**G**	**T**/**C**
Multiread base	**A**	**C**	**G**	**T**
Inference	G to A mutation	G to C mutation and methylated C	No mutation	G to T mutation or G to C mutation and unmethylated C
Prior	Pr(GA)	Pr(GC)xPr(me)	1-Pr(SNP)	Pr(GT)+Pr(GC)x[1-Pr(me)]

Table 1d: Prior probabilities at T reference genome of forward alignments

Reference base	**T**	**T**	**T**	**T**
Unobserved	**A**	**C**	**G**	**T**/**C**
Multiread base	**A**	**C**	**G**	**T**
Inference	T to A mutation	T to C mutation and methylated C	T to G mutation	No mutation or T to C mutation and unmethylated C
Prior	Pr(TA)	Pr(TC)xPr(me)	Pr(TG)	[1-Pr(SNP)]+Pr(TC)x[1-Pr(me)]
