# Supplementary material for: A Bayesian Assignment Method for Ambiguous Bisulfite Short Reads
Source: PLoS One. 2016 Mar 24;11(3):e0151826. doi: 10.1371/journal.pone.0151826 (PMC4806927; doi:10.1371/journal.pone.0151826)
Supplement: S2 Table — (PDF) [file pone.0151826.s006.pdf]

Table 2: Prior probabilities of all possible cases of alignments on the reverse direction

Table 2a: Prior probabilities at A reference genome of reverse alignments

Reference base	**A**	**A**	**A**	**A**
Unobserved	**A/G**	**C**	**G**	**T**
Multiread base	**A**	**C**	**G**	**T**
Inference	No mutation or A to G mutation and unmethylated G	A to C mutation	A to G mutation and methylated G	A to T mutation
Prior	[1-Pr(SNP)]+Pr(AG)x[1-Pr(me)]	Pr(AC)	Pr(AG)xPr(me)	Pr(AT)

Table 2b: Prior probabilities at C reference genome of reverse alignments

Reference base	**C**	**C**	**C**	**C**
Unobserved	**A/G**	**C**	**G**	**T**
Multiread base	**A**	**C**	**G**	**T**
Inference	C to A mutation or C to G mutation and unmethylated G	No mutation	C to G mutation and methylated G	C to T mutation
Prior	Pr(CA)+Pr(CG)x[1-Pr(me)]	1-Pr(SNP)	Pr(CG)xPr(me)	Pr(CT)

Table 2c: Prior probabilities at G reference genome of reverse alignments

Reference base	**G**	**G**	**G**	**G**
Unobserved	**A/G**	**C**	**G**	**T**
Multiread base	**A**	**C**	**G**	**T**
Inference	G to A mutation or no mutation and unmethylated G	G to C mutation	No mutation and methylated G	G to T mutation
Prior	Pr(GA)+[1-Pr(SNP)]x[1-Pr(me)]	Pr(GC)	[1-Pr(SNP)]xPr(me)	Pr(GT)

Table 2d: Prior probabilities at T reference genome of reverse alignments

Reference base	**T**	**T**	**T**	**T**
Unobserved	**A/G**	**C**	**G**	**T**
Multiread base	**A**	**C**	**G**	**T**
Inference	T to A mutation or T to G mutation and unmethylated G	T to C mutation	T to G mutation and methylated G	No mutation
Prior	Pr(TA)+p(TG)x[1-Pr(me)]	Pr(TC)	Pr(TG)xPr(me)	1-Pr(SNP)
